# The biology of imaging

**DOI:** 10.1098/rsta.2020.0389

**Published:** 2022-04-04

**Authors:** Emmanuel G. Reynaud

**Affiliations:** School of Biomolecular and Biomedical Science, University College Dublin, Co. Dublin D04 V1W8, Republic of Ireland

**Keywords:** fluorescence, bioluminescence, history of science

## Abstract

On the cave wall, a discrete but stunning silhouette runs across the uneven surface. Standing still for more than 45 000 years, this is a witness to the ever-enduring need of mankind to image the world around us. The biological world that feeds us is a primary source of inspiration but also an essential element to creating the imaging systems we use every day. But once obscured by the technological jargon and the thunderstorm of numbers and algorithms, those origins fade away into the background. This small piece is about a few marvellous little stories about the biology of imaging, not the debate about the origin of vision and the eye but rather about plants and animals that open the world to new dimensions of biological imaging to fully image the biological world. An eye for an eye.

This article is part of the Theo Murphy meeting issue ‘Super-resolution structured illumination microscopy (part 2)’.

## 1. Four eyes read better than two

Biological imaging is often shrunken to a handful of microscopy techniques mainly using fluorescence while the biological world may span to the far outposts of Universe (e.g. astrobiology) in every shape and size you can think of and probably beyond. This amazing world has been drawn, sculpted, and engraved by mankind for thousands of years using their eyes. But human vision is an imperfect apparatus. It does not see all the colours in the same way; parts wear out and with age issues such as cataracts blur the world. And with manuscripts and knowledge on a page, reading can become difficult with those pesky unreliable eyes. Those forms of bad eyesight are very common. Blurred vision is usually caused by a refractive error one-way (near-sightedness, myopia) or another (far-sightedness, hyperopia) not forgetting my very own astigmatism. But for the ageing reader, presbyopia is the one. The inability to focus light directly on the retina is the foundation of microscopy.

**Figure 1.**
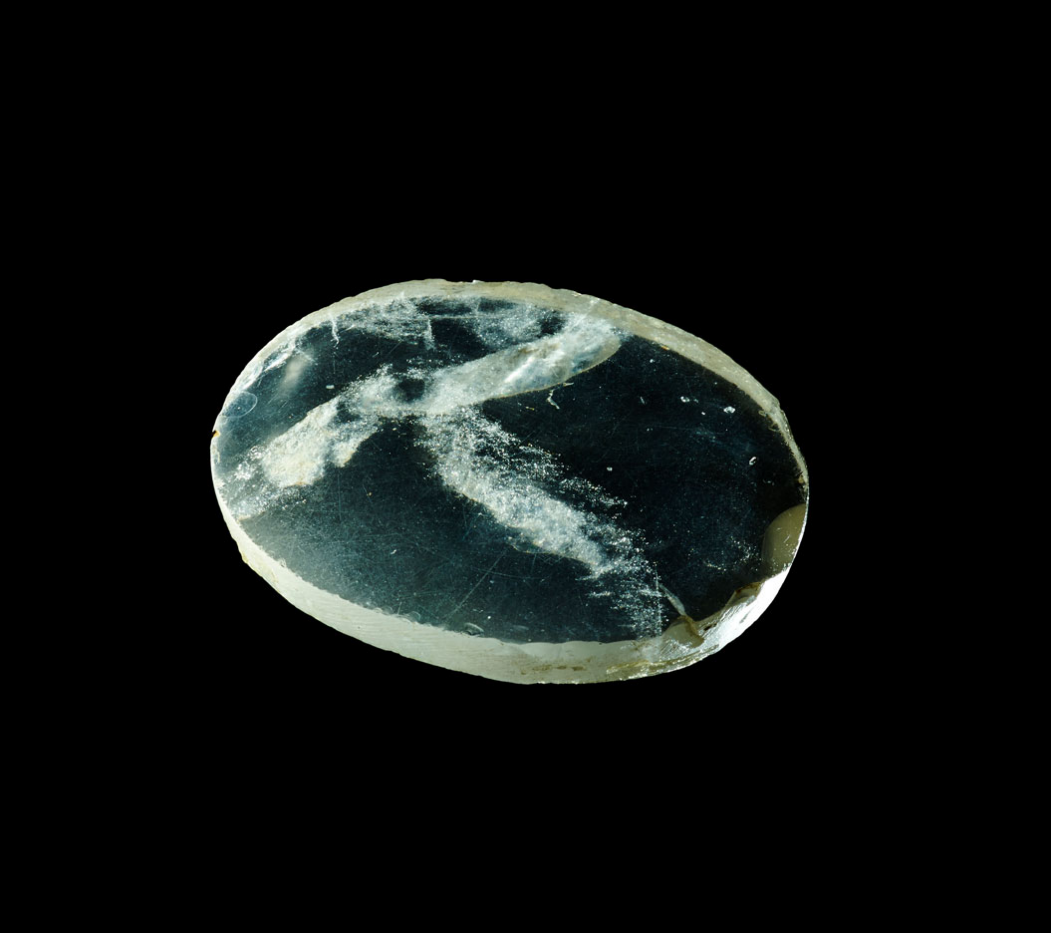
The Nimrud lens, AKA Layard lens, ground polished rock crystal, 750BC–710BC, Neo-Assyrian. (British Museum). (Online version in colour.)

Maybe the Nimrud lens (710 BC), a piece of rock crystal unearthed by Austen Henry Layard at the Assyrian palace of Nimrud in modern-day Iraq, was the first-ever magnifying glass helping an ageing Assyrian scientist to decipher texts. Reading stones, a glass sphere, became common around 1000 AD placed on parchment to magnify the text to aid readability. With population increasing across the world and the evolution of our society allowing us to live beyond the hunter–gatherer view, many genes that will have led to certain death before, survived and travelled with us far and wide including genes affecting vision (figure 1).

Every problem has a solution and around 1284 Salvino D'Armate is credited with the development of the first spectacles or eyeglasses thanks to the flourishing Italian glassblowing industry. Two simple convex lenses were joined together with a central joint linked to a frame made of material such as bone, wood, wire or even leather. They were more convenient to use than reading stones as they could stay on the nose (pince-nez style), allowing reading. And reading pushed the development of eyeglasses and so lenses as Johannes Gutenberg with the invention of the printing press made books widely available. This need to decipher texts and overcome the less than adequate biology of the eye in some pushed the development of better lenses. Zacharias Janssen and his son Hans combined multiple lenses in a tube, the forerunner of not only the compound microscope but also the telescope. Galileo Galilei, in 1609, established the winning combination of a concave and a convex lens in the compound ‘microscope', a term coined in 1625 by Giovanni Paver.

So, it could be said that the origin of microscopy is the combination of an imperfect visual apparatus and a curious nervous system.

## 2. Gin and tonic

**Figure 2.**
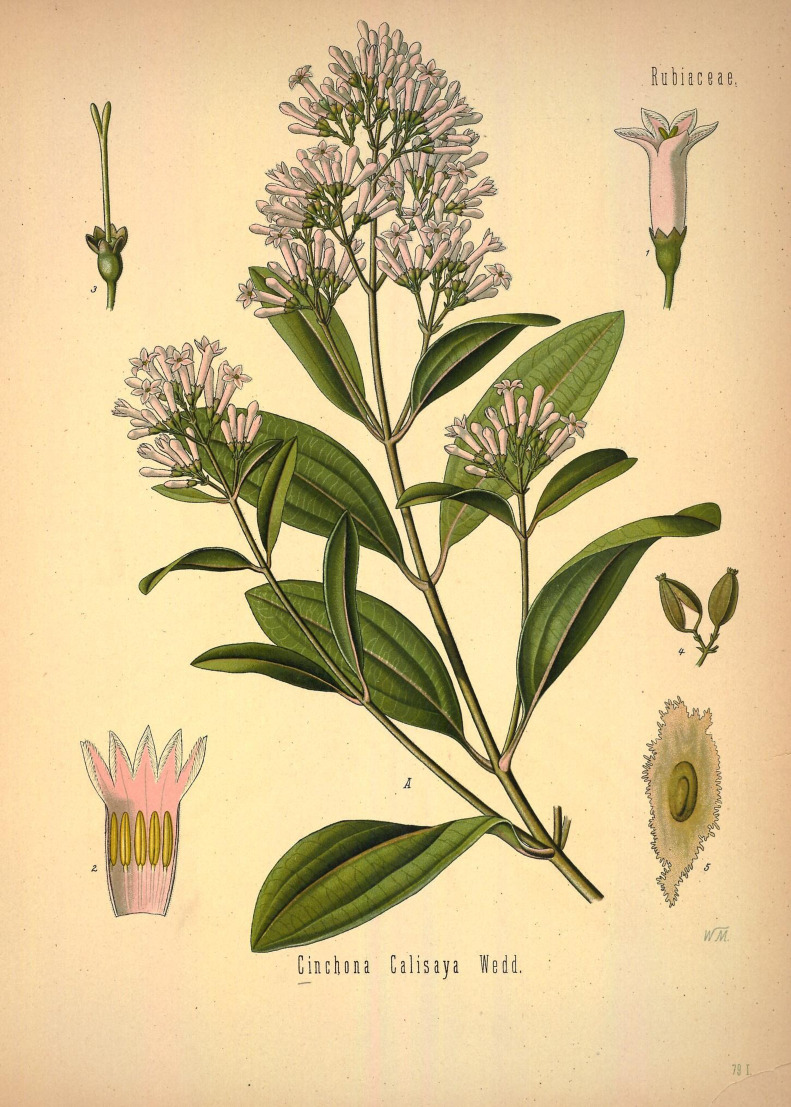
*Cinchona calisaya*. Illustration from Köhler's (not the illumination one) Medizinal-Pflanzen in naturgetreuen Abbildungen mit kurz erläuterndem Texte v. 1, Biodiversity Heritage library [https://www.biodiversitylibrary.org/page/303722]. (Online version in colour.)

Our bad eyesight may have required the development of lenses that are the essential element of any microscope nowadays, but it is another disease that gives us access to a very important modality for optical imaging: fluorescence. *Plasmodium falciparum* and *falciparum vivax* are the main vectors of malaria and have been an integral part of human history for tens of thousands of years across the globe from China to South America. Even though this disease was known in Europe, the treatment was rather rudimentary and cruel, ranging from limb amputation to bloodletting. No real cure was available until an Italian fellow working for a Spanish one tried to find the shortest route to the Spices (Hum! Sounds like a Frank Herbert Dune line) to discover the Americas. Thousands followed including Francisco Pizzaro, the Spanish conquistador. He went deeper into the continent looking for El Dorado and reached Lima, Peru in 1535 after eight years. Growing in the jungle of Peru, not far from the Paddington tribe territory, was a tree; the quina-quina tree, AKA the fever tree. The bark of this tree, cinnamon in colour, when made into a powder and given as a drink could cure the fevers, as an Augustinian monk, Antonio de Calancha, discovered around 1630. This mixture contains several alkaloids including quinine [1]. The remedy made its way to Europe promoted by the Jesuit Order, and it was sometimes called Jesuit's bark or powder. The miracle tree was finally named in 1742, by botanist Carl Linnaeus who called the tree ‘Cinchona' in honour of the Spanish Countess of Chinchon who, while in Peru, contracted a fever that was cured by the bark of a tree (figure 2).

This cure later known as Indian tonic was bitter and soon the British troops based in India found a magical way of tuning it down using gin, a spirit made using another plant, juniper [2]. But this is what, in 1845, Sir Frederik William Herschel did with a solution of quinine that makes our imaging hearts glows. He noted that using certain incidences of sunlight on a normally colourless and transparent quinine solution makes it exhibit a ‘vivid and beautiful celestial blue colour’ [3]. This is the first reported observation of fluorescence. In a quinine solution like tonic water, the ultraviolet light from the sun excites quinine to emit blue light, most apparent when observed at a right angle relative to the incident sunlight. So, raise a gin and tonic glass to a Peruvian tree, a Spanish countess, an English scientist, and a blinking light at the end of a light path! Cheers.

## 3. Thanks for all the jellyfish!

**Figure 3.**
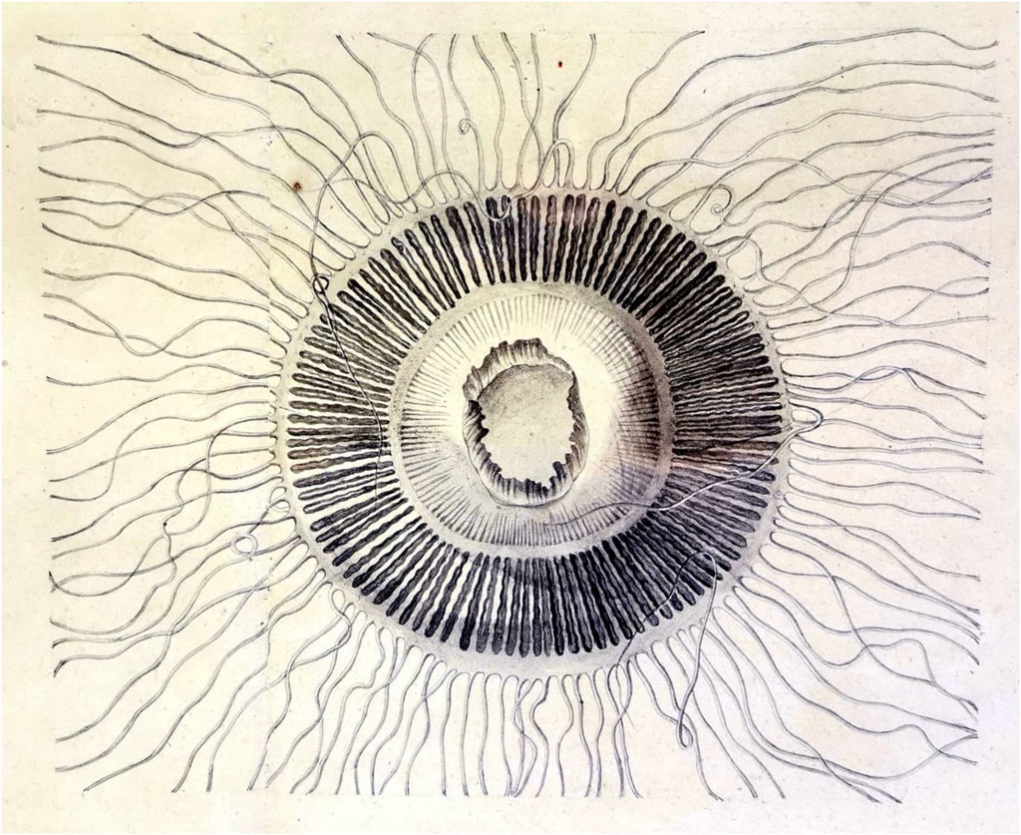
*Aequorea aequorea* by Charles Alexandre Lesueur and Francois Peron. This is the established type use for the *Aequorea* genus [4]. (Online version in colour.)

Charles Alexandre Lesueur, a young French artist, was very excited on the 19th of October 1800 when he boarded ‘Le Naturaliste', one of two vessels which were part of the expedition led by Nicolas Baudin, ordered by Napoleon Bonaparte to explore New Holland (Australia to us). After the death of the expedition's dedicated zoologist René Maugé, he took over the naturalist duties and alongside François Péron collected thousands of zoological specimens including an innocent but beautiful jellyfish that they named *Aequorea* from the Latin, feminine of *aequoreus,* of the sea… (figure 3). They collected several *Aequorea* sp. including *Aequorea phospheriphora* (now *Rhacostoma atlanticum*) and noted: ‘in the midst of the darkness the animal seems surrounded by a circle of fire, gleaming a green, purple and blue light’ [5]. Those little hydrozoan jellyfish, almost entirely transparent, started a long story that has enlightened our cells, embryos and even rabbits. But before claiming victory (Victoria…) and being the most well-known jellyfish in the world, squeezed and shredded to give away her light, it had a bit of a taxonomical journey. Naming species was a tough but important game at the time and with the wealth of new species being discovered and playing empires (British versus French versus Spanish) made taxonomy a bit of a war game.

*Aequorea* [5] was challenged by the mighty Johann Friedrich Gustav von Eschscholtz who decided at the end of his long life as a naturalist to make some room for more species in the phylum Cnidaria where everybody dumped new species by the bucket-load. He created new classes, subclasses, orders, families and finally genera that helped many species find a matching home but *Aequorea* got a new name, *Mesonema*, in 1829. And then the battle went on with *Mesonemanna*, *Mesonemella* and *Mesonemissa* by Ernst Haeckel; *Zygodactyla* by Brandt or *Rhegmatodes* by A. Agassiz which Haeckel quickly renamed *Rhegamatella*. This madness came to an end after almost a century when Louis Murbach and Cresswell Shearer were on a field trip to the coast of British Columbia in the summer of 1900 in the company of Prof. MacBride, of McGill University. They discovered a new species that they could collect in considerable numbers at the entrance of the Victoria Harbour, in Puget sound. And the name was easy to choose; a Mesonema type jellyfish found in Victoria harbour … *Mesonema victoria* … . but the genus was moved back to *Aequorea* a few years later and so we now celebrate *Aequorea victoria* …  the Victory of the Sea, catchy isn't it!

**Figure 4.**
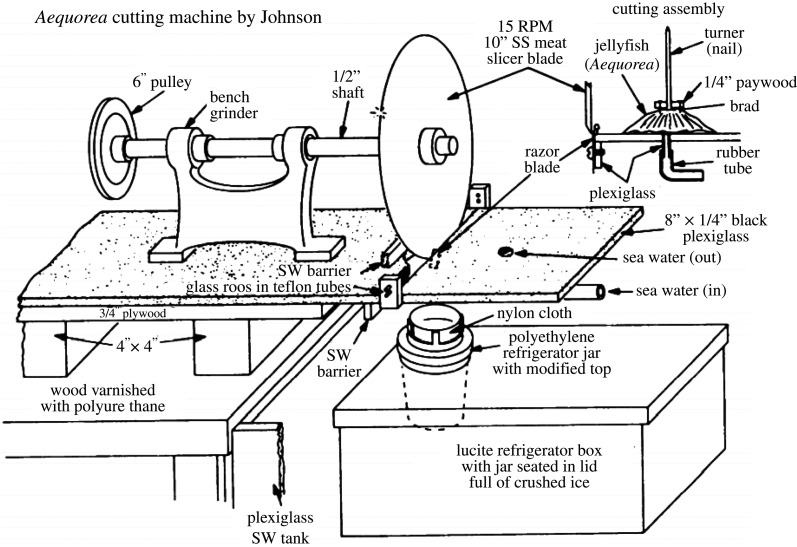
The *Aequorea* cutting machine, designed by F. H. Johnson who was working in Shimomura laboratory, to optimize purification by cutting only the light-emitting rings from the bell. The jellyfish was spun while the disk rotated, the ring was dropping in a dedicated box. (Naval Research Reviews, 1970, 16–23).

While luminescence in medusae was a known fact for nearly two millennia when Pliny in the first century described a luminous slime from the bell of ‘*Pulmo marinus*' (probably *Pelagia Noctiluca*) that could be spread on surfaces making them glow, later studies by the renowned anatomist Spallan (1798) showed that this phenomenon continues after the death of the animal. The understanding of luminescence in invertebrates came to light when the French physiologist Raphael Dubois (1887) decided to ‘juice out' a bunch of *Pholas dactylus* clams, or common piddock, that graced him with light! (see below). The juice idea was not lost as nearly a century later Osamu Shimomura [6] collected bucket loads of *Aequorea victoria* and purified their light-emitting organs through a gauze to obtain what they called the ‘squeezate’ [6]. But Shimomura came about this tiny jellyfish through the bioluminescence route of another unsung hero of our imaging life; the ostracod *Cypridina*. This little creature (2–3 mm long) was well-known in Japan as once collected and dried *Cypridina*, ‘keeps its luminescence property almost permanently, and it will emit light again by simply wetting it with water. Because of this property, the Japanese military collected a large quantity of this organism during the World War II for intended use as a low-intensity light source’ [7]. Shimomura extracted its luciferase and so was ready with all the knowledge necessary to go on and extract from the very dimly luminescent ‘squeezate' what will become the premise of a revolution for us imaging scientists that led to the fluorescent proteins that lit our plates in all shades and colours. Shimomura's laboratory became the birthplace of the GFP and developed countless innovations including the amazing jellyfish trimming machine (figure 4).

## 4. Glow, clam, glow

**Figure 5.**
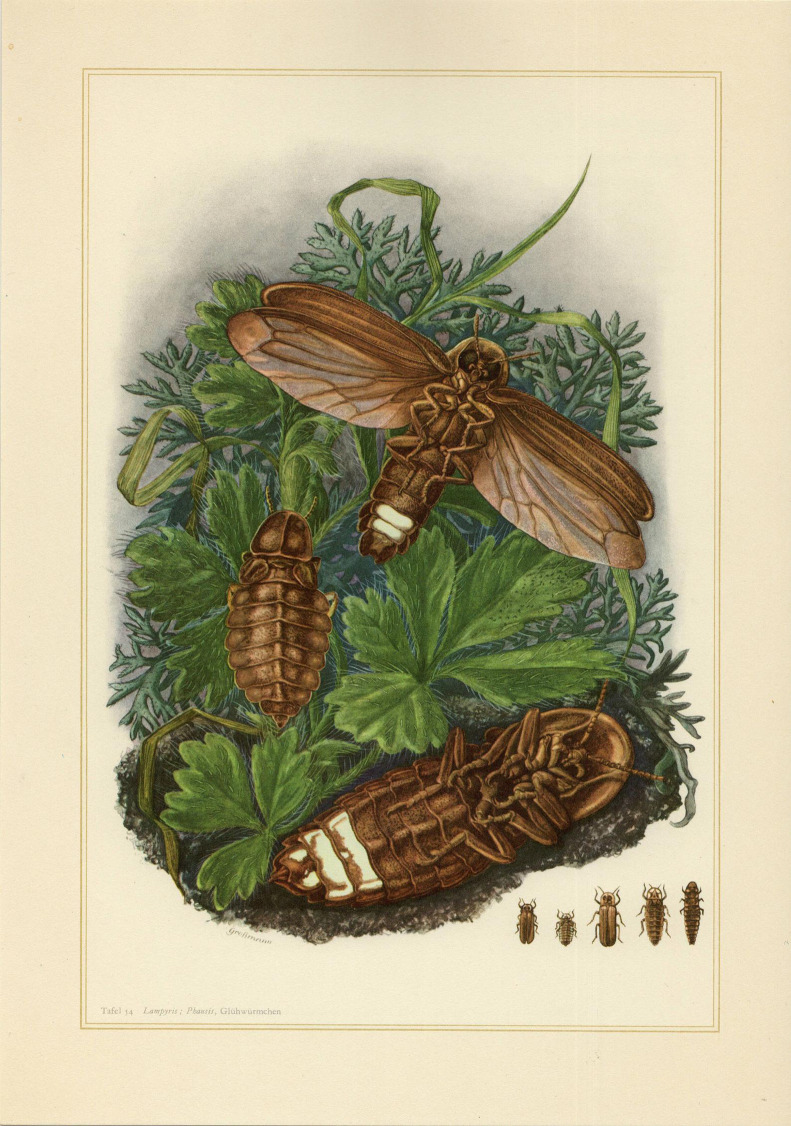
*Lampyris noctiluca*, (firefly) (Linnaeus, 1767). (Online version in colour.)

Sometimes, the imaging scientist crawls out of his dark imaging laboratory late at night after hours of searching for the light, smiling from ear to ear as he finally sees the tiny speckle of green light in the right organelle at the right time, joy! But then, here comes hunger and the scientist rushes for a late-night snack. And will it be cool to get a snack that fits the moment. Pliny, the Roman Statesman, knew the right snack, the piddock (a clam) that would ‘glitter both in the mouth of persons masticating them and, in their hands, and even on the floor and on their clothes when drops fall on them, making it clear beyond all doubt that their juice possesses a property that we should marvel.’ Many people wondered about luminescence and so bioluminescence [8,9]. Conrad Gessner (1516–1565; [10]) published ‘De Lunariis' describing animals including luminous stones and birds. And the view that the act eyes emitted light (Geissner, 1550)! Athanasius Kircher (Love the name!) (1602–1680) was the first to propose that bioluminescence must serve a purpose in those animals and that it only glows for a certain duration…until death! Even Robert Boyle (1627–1691; [11]) focused on the chemistry of rotting wood and fishes finding out along the way that oxygen is a key factor (Boyle, 1665)! Bioluminescence is found not only in juicy clams but in insects, octopuses and dinoflagellates with fascinating names: *Photinus pyralis* (figure 5)*, Pelagia noctiluca, Mycena chloropos, Watanesia scintillans*, as well as *Aequora victoria.* Even the vampire squid, *Vampyroteuthis infernalis*, uses bioluminescent ink to distract his predators while brittle stars will ‘detach’ a glowing body part while the rest of their body crawls away in the darkness. Others like to make a light show in the waves such as the ‘Sea Sparkle’ (AKA ‘Sea Ghost’, ‘Fire of the Sea’, *Noctiluca scintillans*) whose blooms turn every wave into a cascade of mesmerizing blue light, while the lightning bugs will light up for their mates.

But it is French physician Raphaël Dubois (1849–1929), puzzled by the chemiluminescence discovered in 1877, who wondered if bioluminescence could be a chemical reaction and went back to Pliny's luminescent midnight snack; the clam *Pholas dactylus *… He juiced them and played with temperature, mixing cold and heated concoctions and making the heated one glow again when mixed with the cold one! So, a heat stable molecule combined with a heat labile molecule created a glow … The heat stable one was called obviously luciferin, for Lucifer the Light-Bearer, the Light-Bringer … as well as the Latin name for Venus the morning star. And luciferase the enzyme able to deliver Lucifer's light! Since then, many animals, bacteria and fungi have been ‘juiced’ to provide us with countless options in the bioluminescent spectra to image animals while people hope to grow bioluminescent trees to light our streets and bioluminescent crops that will light up when something is wrong or ready to be harvested!

## 5. Be kind, respect other people and biology

The biological world is an amazing world to image thanks to itself and the tools it gave us. But be a biologist, wear your profession with pride and respect taxonomists and other biologists…and spell the bloody species you refer too correctly … . They are Latin … italics, they are binomial *Homo sapiens*, not homo Sapiens … and spend some time learning their genera, family and friends and wonder about the names of some unusual species like the wasp *Aha ha*, the unicellular *Kamera lens* or the well-known sponge *Spongiforma squarepanstsii*. And as Julius Caesar once said: *Vini vidivici* … an extinct parrot!

## Data accessibility

This article has no additional data.
